# Enhanced Phytoextraction Technologies for the Sustainable Remediation of Cadmium-Contaminated Soil Based on Hyperaccumulators—A Review

**DOI:** 10.3390/plants14010115

**Published:** 2025-01-03

**Authors:** Xuerui Cao, Qing Dong, Lihui Mao, Xiaoe Yang, Xiaozi Wang, Qingcheng Zou

**Affiliations:** 1 Zhejiang Institute of Landscape Plants and Flowers, Hangzhou 311251, China; caoxr@zaas.ac.cn (X.C.);; 2 Key Laboratory of Environmental Remediation and Ecological Health, Ministry of Education (MOE), College of Environmental and Resource Sciences, Zhejiang University, Hangzhou 310058, China

**Keywords:** cadmium, hyperaccumulator plants, assisted phytoextraction, soil remediation

## Abstract

Heavy metal pollution in soil is a significant challenge around the world, particularly cadmium (Cd) contamination. In situ phytoextraction and remediation technology, particularly focusing on Cd hyperaccumulator plants, has proven to be an effective method for cleaning Cd-contaminated agricultural lands. However, this strategy is often hindered by a long remediation cycle and low efficiency. To address these limitations, assisted phytoextraction has been proposed as a remediation strategy based on the modification of certain traits of plants or the use of different materials to enhance plant growth and increase metal absorption or bioavailability, ultimately aiming to improve the remediation efficiency of Cd hyperaccumulators. To thoroughly understand the progress of Cd hyperaccumulators in remediating Cd-polluted soils, this review article discusses the germplasm resources and assisted phytoextraction strategies for these plants, including microbial, agronomic measure, chelate, nanotechnology, and CO_2_-assisted phytoextraction, as well as integrated approaches. This review paper critically evaluates and analyzes the numerous approaches and the remediation potential of Cd hyperaccumulators and highlights current challenges and future research directions in this field. The goal is to provide a theoretical framework for the further development and application of Cd pollution remediation technologies in agricultural soils.

## 1. Introduction

In recent years, the acceleration of industrialization has exacerbated soil environmental quality issues, leading to a gradual deterioration of the situation in terms of heavy metal pollution in cultivated land globally. There are over 20 million ha of land contaminated by heavy metal(loid)s worldwide, where the concentration of metals in soil is higher than the regulatory levels or geo-baseline [1]. According to the “Summary of the National Ecological and Environmental Quality in 2020” released by China’s Ministry of Ecology and Environment, Cd is the primary heavy metal contaminant in agricultural land [2]. Cd exhibits non-degradability and persistence once it enters soil from a wide range of sources, representing a major environmental hazard and a public health emergency in many parts of the world. There may be risks to soil elemental balance, microbial community structures, and enzyme activities [3]. Not only does Cd harm soil ecosystems, but it also enters plants via root absorption, interfering with numerous growth processes and adversely affecting overall plant metabolism and reproductive health [4]. Beyond its ecological impacts, the socio-economic ramifications of Cd are even more pressing and far-reaching. In severe cases of Cd contamination, polluted farmland may become unusable for agricultural production, leading to the loss of arable land resources. Such losses affect food security and the sustainable development of agriculture. In particular, Cd could pose potential risks to food safety and human health through the food chain due to its high mobility and bioavailability and may lead to diseases affecting the kidneys, respiratory system, and nervous system [5]. The treatment and rehabilitation of these diseases require substantial medical and social resources, thereby incurring significant medical costs and societal burdens. Given this, implementing effective remediation for Cd-contaminated soil and constructing a green, safe, and sustainable ecological environment have become significant national strategic demands. Currently, the remediation of Cd pollution in farmland soil has emerged as a hot research and practical topic in the field.

Phytoextraction, as a phytoremediation technique, is a promising technology for the remediation of heavy metal-contaminated soils with the advantages of cost-effectiveness, environmental friendliness, and in situ remediation. It is considered suitable for arable land remediation [6] and aligns with ecological civilization construction principles. As ideal materials for Cd phytoextraction, Cd hyperaccumulator plants have attracted significant attention from scholars due to their exceptional ability to absorb, transport, and tolerate Cd in their aerial parts [7] and are widely used in the remediation of Cd-contaminated soils. Therefore, in situ phytoextraction based on Cd hyperaccumulator plants is an effective remediation technology for Cd-polluted farmland soil. However, the low biomass and slow growth of Cd hyperaccumulators pose major obstacles to phytoextraction at a large scale [8]. To achieve higher removal efficiency, assisted measures are fundamental to the practical application of phytoextraction technologies. Consequently, continuous research on enhancement measures for Cd hyperaccumulators in the remediation of Cd contamination in farmland is crucial for promoting the widespread application of phytoextraction technologies. This has inspired researchers to develop additional methodologies known as assisted phytoextraction, which involve alterations of specific plant attributes or the employment of varied substances to enhance plant development and increase metal absorption or bioavailability. Therefore, this paper comprehensively reviews the latest published literature on the germplasm resources and assisted phytoextraction approaches of Cd hyperaccumulators, aiming to provide a comprehensive understanding of the progress in soil Cd pollution remediation using hyperaccumulator plants. This serves as a foundational theoretical framework for advancing the development and application of Cd pollution remediation technologies in farmland soils while also providing vital technical support for the comprehensive prevention, mitigation, and control of Cd pollution in agricultural lands.

## 2. Cd Hyperaccumulator Plants

The concept of hyperaccumulators was first proposed by Professor Brooks et al. [9], specifically referring to plant species growing in natural environments with nickel (Ni) content exceeding 1000 mg kg^−1^ based on dry weight. Currently, the definition of hyperaccumulators has been expanded to encompass plant populations that can actively and efficiently absorb and accumulate one or more trace elements from the soil while exhibiting high tolerance. Screening of hyperaccumulators is a prerequisite for the application of phytoextraction technologies. This process necessitates a scientific and systematic evaluation framework that delves into the plants’ geographical distribution and evolutionary history, as well as transport and accumulation mechanisms of Cd, to identify and confirm novel hyperaccumulators.

To date, over 700 species of hyperaccumulators have been identified globally [10], and this number continues to grow as new species emerge from further research. However, Cd hyperaccumulators are relatively scarce, mainly concentrated within five families [11]. These plants exhibit regional distribution patterns, particularly concentrated in metallic mining areas. In recent years, numerous representative achievements have been made in research on Cd hyperaccumulators both domestically and internationally. Some of these representative discoveries are listed in Table 1, which provides valuable resources for phytoextraction aimed at Cd-contaminated soils. Currently, the primary approaches for screening hyperaccumulators focus on two directions: field investigations in natural environments and indoor simulation studies using soil pots or hydroponic culture. Furthermore, with the rapid advancement of biotechnology, gene engineering techniques have been employed to precisely locate and screen gene fragments closely related to efficient absorption and accumulation of Cd by plants, thereby enhancing plant Cd accumulation capabilities through transgenic technology [12]. It is particularly urgent to accelerate the selection and identification of Cd hyperaccumulator resources. Therefore, we should focus on hyperaccumulators with the potential for large-scale field cultivation and effective Cd pollution mitigation in farmland soils, which is of profound strategic significance in ensuring food security and promoting sustainable agricultural development.

One of the key characteristics of Cd hyperaccumulators is their resistance to technogenic pollution by Cd. These plants have developed mechanisms to tolerate and accumulate high concentrations of Cd without suffering from its toxic effects. A comprehensive understanding of the physiological and molecular mechanisms of hyperaccumulator plants, particularly through the utilization of emerging technologies such as transcriptomics and proteomics, could provide a scientific basis for the remediation of heavy metal pollution and the advancement of phytoextraction technologies. At the physiological level, a robust antioxidant mechanism in the roots is a prerequisite for Cd hyperaccumulator plants to hyperaccumulate Cd. Hyperaccumulators store Cd in specific cellular compartments (such as vacuoles) and reduce its toxicity through chelation, thereby maintaining homeostasis in the aboveground parts [13]. At the molecular level, the unique physiological mechanisms of Cd hyperaccumulators are closely related to the regulatory expression of their genes and proteins. Based on previous transcriptome data, a large number of crucial coding genes related to mineral nutrition, Cd ion transport, and antioxidant enzyme biosynthesis, such as *SaMT3*, *NRAMP*, *HMA*, and *ZIP*, have been identified, and their expression patterns under different Cd stress conditions have been regulated [14,15]. These genes may encode proteins involved in the absorption, transport, storage, and detoxification of Cd. Proteomics-based approaches, by identifying Cd-induced differentially abundant proteins (DAPs), contribute to a deeper understanding of plant responses to Cd stress at the systemic level. The enhanced DAPs discovered through proteomics research can serve as potential targets for Cd hyperaccumulation that can be utilized for the remediation of Cd-contaminated soils [16].

Genetic variability is a fundamental aspect of biological populations, enabling them to adapt to changing environments and resist various stresses. In the context of hyperaccumulators, genetic variability within populations determines the diversity of traits related to heavy metal absorption, translocation, and tolerance. This variability serves as a valuable resource for genetic breeding efforts aimed at developing high-efficiency and resilient hyperaccumulator varieties. However, the introduction of non-native hyperaccumulator plants and genetically modified organisms (GMOs) could pose potential threats to existing ecosystem structures, biodiversity, and the Earth’s environment. They may proliferate extensively after seizing environmental resources, leading to the displacement of “native” species in their habitats. This, in turn, can result in a trend towards species homogenization, a decline in the number of biological species, gradual loss of biodiversity, and imbalance in ecosystem structures. Additionally, GMOs may cause gene flow and genetic pollution, further endangering ecosystems [17]. Therefore, the introduction must be carefully managed to minimize potential ecological risks by conducting thorough risk assessments, close monitoring, and the implementation of effective management strategies in order to ensure the sustainability and health of our ecosystems.

**Table 1:** Representative Cd hyperaccumulator plants.

Cd Hyperaccumulator (Family)	Discovery Site	Cd Concentration in Ground (mg kg^−1^)	Methods	References
*Thlaspi caerulescens* L. (Brassicaceae)	Metalliferous sites from the north and south Pennine orefields	14,187	Field collection and solution culture	[18,19]
*Sedum alfredii* Hance (Crassulaceae)	An old Zn/Pb mining area in Quzhou, China	6500 (Shoot) 9000 (Leaf)	Field investigation and solution culture	[20]
*Sedum plumbizincicola* (Crassulaceae)	A Zn/Pb mining area in Zhejiang, China	587	Field investigation and solution culture	[21]
*Viola baoshanensis* (Violaceae)	Baoshan mining area in Chenzhou City, Hunan Province, China	2310	Field investigation and greenhouse experiment	[22]
*Solanum* *nigrum* L. (Solanaceae)	Around Shenyang Ecological Experiment Station, Chinese Academy of Sciences	103.8 (Shoot) 124.6 (Leaf)	Pot and field experiment	[23]
*Arabis* *paniculate* L. (Brassicaceae)	Jinding Pb/Zn mining area, Lanping County, Yunnan Province, China	3569	Field investigation and solution culture	[24]
*Aster subulatus* Michx (Asteraceae)	A Pb/Zn mining area in Guangxi, China	5672.5 (Shoot) 862.5 (Leaf)	Field investigation and sand-based solution culture	[25]

## 3. Strategies for Assisted Phytoextraction Based on Cd Hyperaccumulators

**Figure 1:**
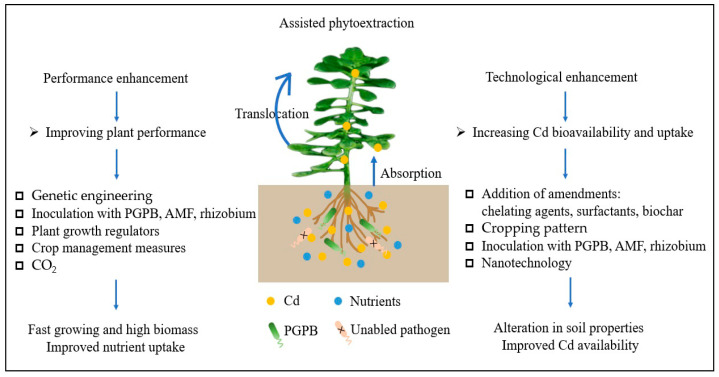
Enhancement strategies for the assisted phytoextraction of Cd for remediation.

Despite some progress made in Cd hyperaccumulator research in recent years, large-scale applications still remain constrained due to their low biomass, slow growth, and lack of suitable mechanized operations. Currently, the hyperaccumulators discovered and applied to the remediation of Cd-contaminated farmland soils are mainly concentrated in the Crassulaceae family. It is fundamental for the practical application of phytoextraction to enhance the remediation efficiency of plants through intensified measures. Therefore, we must continue to strengthen research on enhancement measures for Cd hyperaccumulators in the remediation of Cd-contaminated arable lands, namely assisted phytoextraction. Assisted phytoextraction could enhance Cd phytoextraction, mainly by improving plant performance and increasing Cd bioavailability and uptake. Therefore, such methods are divided into performance enhancement and technological enhancement techniques based on the differences in enhancement methods and mechanisms (Figure 1). Performance enhancement refers to the improvement of the remediation capabilities of plants themselves through crop breeding techniques or the application of microbial agents. Technological enhancement refers to the indirect increase in the remediation efficiency of plants by activating Cd in soil that is difficult for plants to absorb. The primary assisted phytoextraction studies have included microbial, agronomic, chelate, nanotechnology, and CO_2_ measures, as well as integrated enhancement approaches.

### 3.1. Microbially Assisted Phytoextraction

Microbial augmentation has been extensively applied in the remediation of Cd-contaminated soils due to its advantages of low risk, cost-effectiveness, and environmental friendliness. Under long-term heavy metal contamination, plants adapt to the environment by altering their rhizosphere and endophytic microorganisms, resulting in the emergence of specific microorganisms. These specific microorganisms have attracted extensive attention from researchers due to their ability to promote plant growth, enhance plant tolerance to heavy metals, and improve heavy metal absorption [26]. Enhancing phytoextraction efficiency through the coexistence of microorganisms and plants is an active research area, and the effects and mechanisms of microbial regulation on phytoextraction have become hot topics in the field in recent years. Previous studies showed that the application of Bacillus strains QX8 and QX13 increased the biomass of *S. nigrum* stems by 136–170% and Cd concentrations by 228–281% [27]. Inoculation with SaMR12 alleviated Cd stress by reducing the hydrogen peroxide concentration and increasing glutathione levels, ensuring the normal growth of *S. alfredii* under Cd stress and enhancing its Cd absorption and accumulation [28]. Inoculation with Bacillus megaterium NCT-2 elevated the Cd concentration and chlorophyll content in leaves simultaneously, thereby promoting the growth of *S. nigrum* [29]. These microorganisms exhibited plant growth-promoting traits, including siderophore production, auxin synthesis, phosphate solubilization, biological nitrogen fixation, and ACC deaminase activity, which directly or indirectly stimulate plant growth [30]. Consequently, they are also referred to as plant growth-promoting bacteria (PGPB). PGPB have garnered increasing attention due to their ability to enhance plant growth and heavy metal absorption by modulating physiological processes and metabolic pathways, enhancing nutrient uptake, and safeguarding against pathogenic microorganism invasion [31,32,33]. Inoculation with PGPB could increase plant biomass and heavy metal absorption and accumulation [34,35]. Chen et al. (2024) [7] reported that foliar application of *Pseudomonas fluorescens* (a PGPB) significantly increased the biomass of *S. alfredii* by 39.2% and Cd removal efficiency by 106.8%. Wu et al. (2018) [36] studied the effects of a novel endophytic bacterium named Buttiauxella sp. SaSR13 (SaSR13), which was isolated from the roots of Cd hyperaccumulator *S. alfredii*, on plant growth and Cd accumulation. Laser scanning confocal microscopic images showed that SaSR13 primarily colonized the elongation and maturation zones of roots. Under treatment with SaSR13 inoculation, the biomass of both stems and roots significantly increased by 39% and 42%, respectively. Additionally, chlorophyll content was increased by 38%, and Cd concentrations in stems and roots were increased by 32% and 22%, respectively. SaSR13 promoted root development (increased root length, surface area, and the number of root tips) by increasing the indole-3-acetic acid (IAA) concentration and reducing the superoxide anion (O^2−^) concentration under Cd stress conditions. Furthermore, inoculation with SaSR13 stimulated the release of root exudates, particularly malic and oxalic acids, which may facilitate Cd absorption by *S. alfredii*. Chi et al. (2023) [37] isolated an endophytic bacterium, Pseudomonas E3, from Cd hyperaccumulator *S. nigrum* and applied it to the plants. The results revealed that plant biomass increased by 20.47% and Cd extraction efficiency significantly increased by 40.26%. Strain E3 influenced Cd accumulation in *S. nigrum* by regulating the absorption and transport of iron and magnesium at the transcriptional level while maintaining the balance of the photosynthetic system by regulating leaf amylase activity. Additionally, strain E3 recruited specific bacteria (e.g., *Pseudonocardiaceae* and *Halomonas*) to promote plant growth. These two aspects may potentially contribute to the enhanced Cd extraction efficiency observed in *S. nigrum*.

PGPB encompasses not only endophytic PGPB that colonize plant cells or intercellular spaces to elicit physiological responses but also rhizosphere PGPB that colonize plant roots or rhizospheres, interacting with plants [30,38]. Huang (2011) [39] demonstrated that the application of rhizospheric Pseudomonas LKS06 significantly increased Cd accumulation in the aerial parts of *S. nigrum* by 42.4%, significantly enhancing Cd enrichment capacity while alleviating Cd-induced stress. Liu et al. (2015) [40] conducted pot experiments to study the effects of isolated rhizospheric PGPB on the growth and Cd accumulation of *S. plumbizincicola* and found that inoculation with LCR1 strains significantly promoted plant growth and Cd absorption and accumulation. Compared to controls, LCR1 inoculations did not significantly affect Cd concentrations in stems among treatments while significantly increasing the dry weight of the stem by 26.9%. Thus, total Cd accumulation in stems increased by 18%. In another study, inoculation with rhizospheric Rhodococcus NSX2 had no significant effect on *S. plumbizincicola* growth but significantly increased the Cd concentration in stems by 21.6% and total chlorophyll content in leaves, leading to increased Cd accumulation in the stem [41]. These strains exhibited varying levels of IAA production and phosphate solubilization, and rhizospheric PGPB could alter soil microbial community structure and diversity. The impacts of PGPB on phytoextraction can be summarized in two key aspects. First, PGPB could promote plant growth and increase biomass by secreting siderophores, synthesizing plant hormones, performing biological nitrogen fixation, solubilizing phosphorus, producing ACC deaminase, and enhancing stress resistance directly or indirectly. Secondly, PGPB could activate heavy metals in soil via the production of phytochelatins, organic acids, metal chelates, biosurfactants, and redox reactions, thereby facilitating the absorption of heavy metals by plants. The interplay of these two aspects could significantly improve plant heavy metal accumulation and enhance phytoextraction efficiency [38].

Apart from PGPB, there are two other important microbial groups commonly used as carriers for enhanced bioremediation: Rhizobium and arbuscular mycorrhizal fungi (AMF). Sahito et al. (2022) [42] established a root proliferation system facilitated by various Rhizobium strains in Sedum species, and five potential strains (LBA9402, K599, AS12556, MSU440, and C58C1) were identified. Among them, Rhizobium AS12556 promoted root proliferation, increased stem biomass, reduced oxidative damage, and enhanced Cd extraction efficiency in *S. alfredii* by producing phytochemicals exogenously. Therefore, it is considered a potential microbial partner for the development of extensive root systems to promote growth and enhance phytoextraction potential. Liu et al. (2015) [43] studied the effects of AMF on the growth and Cd accumulation of Cd hyperaccumulator *S. nigrum* in soils with varying Cd concentrations in greenhouse pot experiments. Across all Cd levels, AMF-inoculated plants exhibited generally higher mycorrhizal colonization rates (71% to 82%). AMF colonization enhanced soil acid phosphatase activity, which, in turn, improved phosphorus acquisition and the growth of *S. nigrum* under different Cd levels. Compared to uninoculated plants, AMF-inoculated *S. nigrum* showed significantly increased biomass (33–323% for aerial parts and 46–1105% for roots) and total Cd absorption in plant tissues under different Cd levels. AMF could alter the rhizospheric soil microenvironment and regulate specific physiological and biochemical processes in plants, such as chelation, transport, and antioxidant defense systems, thereby promoting plant resistance to Cd stress. Additionally, AMF could promote plant growth to resist Cd stress. 

Most previous studies have focused on the effects of a single PGPB. However, there has been increasing interest in mixed PGPB inoculants in recent years. In a study by Cao et al. (2024) [44], four PGPB strains, namely *Sphingomonas* sp. SaMR12, *Burkholderia* sp. SaZR4, *Variovorax* sp. SaNR1, and *Burkholderia* sp. SaMR10, were combined into a consortium and used as microbial agents in field trials conducted in three locations. The combined strains increased the shoot dry weight of *S. alfredii* by 3.79–22.9% compared to single-strain inoculation. They also slightly elevated the shoot Cd concentration and enhanced Cd accumulation in the shoots to varying degrees across the three sites. Multi-strain PGPB inoculation is a frontier in current plant biofortification due to its broader range of effects and higher environmental adaptability [45].

Recent developments in synthetic biology have transformed the capacity to craft microorganisms with targeted traits, unlocking new possibilities for plant–microbe interactions. Genetic engineering and novel synthetic biology methods are accelerating phytoextraction progress. Genetically engineered microorganisms (GEMs) have emerged as a desired requirement for enhanced bioremediation of HM-polluted soils, offering eco-friendly, cost-effective solutions compared to traditional bioaugmentation [46,47]. Specific heavy metal-binding genes, like *MerR*, *synHMB*, *LECBP*, and *PtMT2b*, were designed and expressed in engineered microbes and plants to promote metal capture and transfer [48,49]. Integrating synthetic biology into the design of PGPB for specific hyperaccumulators is a groundbreaking method that promises to advance phytoextraction. However, the environmental application of genetically engineered organisms has potential risks that need to be avoided.

In summary, the combined use of microbes and plants for remediation is an effective and environmentally friendly approach. However, the success of this method relies heavily on the presence and activity of specific microorganisms, as well as the maintenance of soil conditions that can enhance plant metal absorption and translocation [50]. It is important to note that in diverse soil environments, the composition and function of microbial communities may vary significantly, leading to inconsistent results and limited reproducibility. Another significant concern is the interactions between introduced microbes and native soil microbiota, which are crucial for optimizing the effectiveness of microbially assisted phytoextraction. Moreover, scaling up microbially assisted phytoextraction from laboratory-scale experiments to large-scale field applications could pose significant challenges. These include issues related to the production, storage, and application of large quantities of microorganisms, as well as the need for cost-effective and environmentally friendly methods for their delivery to the soil. Additionally, the introduction of non-native microbial species to enhance metal absorption may pose ecological risks, including potential invasion and disruption of native microbial communities. These limitations underscore the need for thorough assessment and further research to optimize the use of plant-associated bacteria and fungi for safe and effective application in plant phytoextraction.

### 3.2. Agronomic Measure-Assisted Phytoextraction

Plant performance and Cd accumulation are also indirectly stimulated by agronomic measures. The contamination of farmland soils by Cd has resulted in reduced crop yields and potential food security issues. The direct use of hyperaccumulator plants for soil remediation disrupts agricultural production and fails to meet farmers’ regular farming needs. Intercropping has been widely adopted due to its unique advantages. The complex interactions among plants, root exudates, and microorganisms in intercropping systems could affect metal absorption, which has garnered increasing interest from researchers in Cd phytoextraction [51]. Moreover, among the sites with the highest rates of Cd contamination, most are moderately to mildly polluted. Intercropping hyperaccumulator plants with crops could reduce Cd pollution in the soil and produce agricultural products that meet safety standards. Intercropping crops with hyperaccumulator plants may be considered an innovative approach to sustainable agriculture, providing double chances for contaminated soil remediation and agricultural production, which is crucial for countries with limited arable land.

Current research on the safety of crop production and soil remediation effects under Cd hyperaccumulator–crop intercropping systems has shown inconsistent findings. Different crops have led to varying overall remediation outcomes. Ng et al. (2023) [52] explored the feasibility of intercropping Cd hyperaccumulator *S. alfredii* with *Pinellia ternata* to enhance yield and quality under two soil Cd contamination levels. Under low Cd contamination, intercropped *P. ternata* showed 10% and 90% increases in bulb and bulbil yields, respectively, while Cd content decreased by 3% and 12%, respectively, compared to sole *P. ternata*. This indicates that intercropping *S. alfredii* with *P. ternata* enhanced the yield of the herbaceous plant and reduced Cd content in medicinal organs. In another study on the remediation of Cd-contaminated soil, the intercropping of low-Cd-accumulating flowering Chinese cabbage (*Brassica campestris* ssp. chinensis var. utilis) with *S. alfredii* significantly increased the biomass of both plants. Specifically, the Cd content in the shoots and roots of *S. alfredii* increased by 7.61% and 70.73%, respectively, compared to sole cropping. In contrast, the Cd content in the shoots of flowering Chinese cabbage decreased by 27.21%. The soil Cd extraction rate of intercropped *S. alfredii* was 1.88 times higher than that of sole cropping. This intercropping system not only improved the safety of Chinese cabbage production but also enhanced Cd extraction efficiency in *S. alfredii* from the soil. This presents an economical, effective, and lower risk approach to simultaneous production and remediation [53]. For economic crops like oilseed rape, fava bean, and corn, intercropping with *S. alfredii* could increase metal accumulation by 11.5–268% in *S. alfredii* and 2.5–322% in these economic crops while ensuring safe production simultaneously as compared to monoculture [6,54,55]. Moreover, intercropping *S. alfredii* with oilseed rape increased the plant’s biomass and Cd accumulation, regardless of soil type [6]. Hyperaccumulator plants intercropped with crops could effectively promote plant or crop growth and enhance their Cd extraction efficiency while fully utilizing space, light energy, and soil fertility. Moreover, intercropping with suitable crops ensures the safe production of agricultural products, achieving a win–win outcome.

Xu et al. (2024) [56] conducted a relay cropping experiment in Cd-contaminated farmland using four Cd hyperaccumulator plants (*S. alfredii*, *S. plumbizincicola*, *S. nigrum*, and *Amaranthus mangostanus*) with soybeans, oilseed rape, and wheat. They discovered that relay cropping between hyperaccumulator plants with crops significantly increased the biomass of oilseed rape and wheat while reducing the Cd content in the crops. Compared to sole soybean cropping, relay cropping with *S. plumbizincicola*, *S. alfredii*, and *S. nigrum* significantly improved soil Cd removal rates by 82.8%, 64.0%, and 70.4%, respectively. Additionally, relay cropping with *S. nigrum* significantly reduced Cd content in soybean stems and leaves. Deng (2015) [57] found that relay cropping *S. plumbizincicola* with corn significantly improved plant phytoextraction and remediation efficiency through eight consecutive years of field experiments, and the Cd content in corn kernels was below the national food safety limit, meeting safe consumption standards. Under relay cropping conditions, root exudates and metabolites from hyperaccumulator plants can diffuse to neighboring plants, influencing each other through “rhizosphere communication”. This alters the speciation and bioavailability of heavy metals in the rhizosphere, thereby enhancing Cd availability in the soil and promoting plant Cd absorption [58]. Additionally, the root exudates and metabolites of hyperaccumulator plants provide carbon and nitrogen sources for rhizosphere microorganisms, boosting microbial activity and enhancing plant remediation of heavy metal-contaminated farmland soils [59]. Furthermore, the synergistic effects between relay and intercropped plants promote plant growth and increase their Cd absorption and accumulation.

Currently, there are relatively few reports on the rotation of hyperaccumulator plants with common crops. A pot experiment was conducted by Shen et al. (2010) [60] to examine the rotation of hyperaccumulator *S. plumbizincicola* with the low-Cd-accumulating rice variety Zhongxiang 1 in heavy metal-contaminated soil. The study found that planting *S. plumbizincicola* increased the Cd concentration in the shoots of subsequent rice crops and the ammonium acetate-extractable Cd content in the soil. However, the cultivation of *S. plumbizincicola* significantly benefited rice growth. In the rotation of *S. plumbizincicola* with *A. mangostanus*, the preceding *S. plumbizincicola* did not affect the biomass or shoot Cd content of subsequent *A. mangostanus* significantly, but it significantly influenced soil pH and ammonium acetate-extractable Cd content [61]. In a study on the rotation of rice, oilseed sunflower, and *S. plumbizincicola* with varying Cd absorption characteristics, it was found that the highest level of Cd removal occurred in the rotation of *S. plumbizincicola* with high-Cd-accumulating late rice (150.34 g hm^−2^). This was followed by the rotation of *S. plumbizincicola* with oilseed sunflower and conventional early–late rice rotation. After harvesting two crops, the soil Cd content under the *S. plumbizincicola* –high-Cd-accumulating late rice rotation was significantly lower than that under other treatments, indicating that this rotation mode is more conducive to phytoextraction and remediation by *S. plumbizincicola* [62]. Tang et al. (2023) [63] established two rotation modes in moderately Cd-contaminated farmland to investigate their long-term remediation effects on soil Cd and feasibility for safe production. The theoretical soil Cd remediation efficiency under the oilseed rape–sesame–*S. alfredii* rotation mode was 6.74%, which was 1.76 times higher than that under the oilseed rape–peanut–flax rotation mode, demonstrating better Cd contamination remediation.

Although most studies have showed that relay cropping, intercropping, and rotation with hyperaccumulator plants could improve plant Cd remediation efficiency, their effects on individual plant biomass and shoot Cd content varied differently. This variation may be closely related to planting density; soil type; and, more crucially, suitable companion plants, nutrient competition, and growth periods between plants. Overall, each cultivation method has its unique advantages and limitations, primarily influenced by soil type and climatic conditions, as well as plant species (Table 2). When choosing between these patterns, farmers should consider their local soil and climatic conditions, along with their specific crop needs and goals, to determine which method is more suitable.

Apart from cropping patterns, crop cultivation and management techniques based on fertilization, organic materials, water content, planting density, and harvest time may all impact the Cd extraction efficiency of hyperaccumulator plants. Fertilization is a crucial agronomic measure to improve soil fertility and increase crop yield. Numerous studies have shown that fertilization serves as an important auxiliary measure to enhance the phytoextraction efficiency of contaminated soil by hyperaccumulator plants. Increased application of potassium (K) fertilizer is a primary factor in increasing the Cd concentration and accumulation of the shoots of *S. plumbizincicola*. Specifically, high-dose K treatment increased the Cd concentration in the shoots of *S. plumbizincicola* by 28.1% compared to non-K treatment. Enhanced application of nitrogen (N) fertilizer is the primary factor in increasing the shoot dry weight of *S. plumbizincicola*. As N application levels increased, the shoot biomass of *S. plumbizincicola* significantly improved [66]. Yao (2015) [67] investigated the effects of adding different organic materials on the growth of *S. alfredii*. They found that the addition of bamboo shoots was more favorable for Cd accumulation in the shoots of *S. alfredii*, exceeding the control by 12.9%. Yu et al. (2023) [68] studied the influence of soil moisture on phenotypic traits, photosynthetic efficiency, metabolic profile, and Cd accumulation of hyperaccumulator *S. alfredii*. They discovered that different water potential treatments significantly affected plant growth and Cd absorption efficiency. Excessively high or low soil water content could significantly reduce Cd accumulation in the shoots as compared to the ideal water content for *S. alfredii* growth. *S. alfredii* achieved the highest growth rate and yield when soil moisture was maintained at −15 to −30 kPa. When the soil was relatively dry (−45 to −90 kPa), the growth rate and yield of *S. alfredii* gradually decreased. *S. alfredii* could tolerate mild drought stress and achieve maximum Cd absorption within a soil water potential range of −15 to −45 kPa, but it may not withstand soil saturation or flooding conditions. When planted at 450,000 plants per hectare, *S. alfredii* achieved the highest shoot dry matter content and total soil Cd remediation efficiency, which were 1576.0 kg ha^−1^ and 12.8%, respectively. These values were 20.4% and 61.6% higher than those achieved under medium-density planting (300,000 plants per hectare) [69]. Ji et al. (2010) [70] demonstrated that selecting the optimal harvest time helps to increase Cd extraction by *S. nigrum*.

### 3.3. Chelate-Assisted Phytoextraction

Chelate-assisted phytoextraction (the addition of exogenous substances) was shown to affect Cd accumulation and translocation in plants by changing the chemical properties of the soil, including the adjustment of soil acidity and alkalinity, cation exchange, redox potential, and other chemical properties. It could also directly change their form with the combination of heavy metals, thereby enhancing heavy metal uptake by plants [71]. In the context of assisted extraction and remediation using Cd hyperaccumulators, chemical intensifiers including chelators, surfactants, plant growth regulators, and biochar are popular and commonly used. These additives could form stable complexes with Cd in soil or increase its solubility, enhancing Cd bioavailability and making it more readily available for plant uptake.

There are two common types of chelators: synthetic chelators (e.g., EDTA, HEDTA, DTPA, EGTA, NTA, EDDHA, CDTA, and EDDS) and natural chelators (e.g., citric acid, oxalic acid, tartaric acid, and other low-molecular-weight organic acids). Wang et al. (2014) [72] conducted a study using EGTA as an alternative to EDTA to assess its effectiveness and its potential to reduce Cd leaching risks compared to EDTA. The results indicated that both EDTA and EGTA treatments inhibited the growth of *Mirabilis jalapa* L. and significantly increased Cd concentrations in the plants. It was found that EGTA treatment had a greater ability to translocate Cd than EDTA, suggesting that EGTA was more effective in promoting Cd extraction in *M. jalapa* L. However, it is important to note that most synthetic chelators are non-degradable and potentially toxic to the environment, so their usage should be cautiously approached. The use of biodegradable chelators to aid in plant phytoextraction is an effective method for improving the efficiency of heavy metal remediation. One of the main benefits of using organic acids as additives is their ability to biodegrade, which reduces the risk of residual contamination and leaching after the remediation process. In a study involving *S. alfredii* Hance, it was found that adding 1 mg kg^−1^ and 2.5 mg kg^−1^ of oxalic acid to the soil helped to mitigate heavy metal toxicity and significantly increased the stem biomass of the plant. In comparison to the control group, the absorption of Cd increased by 21.5% and 39.1%, respectively, under the two different concentrations of oxalic acid [73]. Under hydroponic conditions, both citric and tartaric acids significantly improved the capacity of *S. alfredii* Hance to absorb and accumulate Cd [74]. Cyclodextrins (CDs) are non-toxic and biodegradable surfactants used to remove hydrophobic compounds from contaminated soils. They can form inclusion complexes with various organic compounds in both solution and solid states, thereby increasing their water solubility [75,76]. Among them, β-cyclodextrin (β-CD) has been successfully applied to enhance Cd absorption in *S. nigrum*. Adding β-CD significantly increased the aboveground biomass and Cd accumulation of *S. nigrum* [77]. Plant growth regulators such as brassinosteroids (BRs), auxins, abscisic acid, ethylene, and others exhibit similar physiological and biological effects to plant hormones and play crucial roles in regulating various plant growth processes and responses to abiotic stresses. Indole-3-acetic acid (IAA) is the most abundant natural auxin, with numerous regulatory functions, including promoting cell division and plumule elongation in plant growth. Auxins could stimulate the absorption of fast-acting nutrients (K and Ca) and have been proven to enhance Cd phytoextraction by inducing positive physiological responses and mitigating Cd stress [78]. Spraying either IAA or GA3 (gibberellic acid) alone promoted the growth of *S. alfredii* Hance. The presence of both IAA and GA3 increased leaf chlorophyll a, carotenoid, and potassium contents while reducing the levels of malondialdehyde. Compared to the control, both IAA and GA3 treatments significantly increased the accumulation of Cd in *S. alfredii* Hance [79]. In recent years, biochar has also been one of the most discussed areas of research. Modified biochar (residual solids from biomass pyrolysis) has been shown to enhance metal phytoextraction from contaminated soils with hyperaccumulator plants under specific conditions. Adding biochar did not significantly affect the Cd content in the aboveground portion of *N. caerulescens* but benefited plant growth and root development, resulting in a 20% increase in the total amount of Cd extracted by plants. Additionally, biochar improved water retention in topsoil and limited nitrogen leaching [80].

The application of chelate enhancement measures could greatly improve the efficiency of Cd removal from contaminated soil by Cd hyperaccumulators. This provides an effective and practical solution for remediating Cd-contaminated soil. However, using excessively high concentrations of stimulators may increase the toxicity of heavy metals in the soil and be harmful to plants. Therefore, it is important to carefully control the dosage concentrations and choose the appropriate timing for application. While many researchers worldwide have made progress in the theory and application of chemical stimulators for phytoextraction of Cd-contaminated soil, further research is needed to develop new environmentally friendly stimulators that degrade quickly, specifically target the metals, leave no residues, and are cost-effective to advance this technology.

### 3.4. Nanotechnology and CO_2_-Assisted Phytoextraction

The application of nanotechnology in the phytoextraction of heavy metal-contaminated soils has also emerged as one of the most discussed areas of research in recent years and is considered a promising technology. Research has shown that molybdenum trioxide nanoparticles (MoO_3_ NPs) could enhance the productive performance of hyperaccumulator *S. nigrum* and promote phytoextraction by stimulating carbohydrate biosynthesis, energy metabolism, and potential succinate signaling defense pathways. Under treatment with MoO_3_ NPs-40, Cd content increased by 1.85 times [81]. Bakshi and Kumar (2023) [82] evaluated the feasibility of using titanium dioxide nanoparticles (TiO_2_ NPs) at different concentrations (0, 100, 250, and 500 mg kg^−1^) in combination with hyperaccumulator *Brassica juncea* L. to effectively remove Cd from soils. The addition of TiO_2_ NPs to soil with a Cd concentration of 10 mg kg^−1^ significantly improved plant photosynthetic activity, leading to accelerated overall plant growth. It also enhanced Cd tolerance and increased the biomass of *B. juncea*, thereby improving soil remediation efficiency.

Studies have indicated that the increasing concentration of CO_2_ in the atmosphere not only affects plant growth but also has significant effects on the absorption and accumulation of heavy metals in plants. In recent years, significant progress has been made in research on Cd hyperaccumulator plants, especially in improving their ability to extract Cd from the soil through CO_2_ fertilization methods. Tang et al. (2019) [83] found that both CO_2_ treatment and endophyte inoculation notably promoted the growth of *S. alfredii* (*p* < 0.05), improved root morphological characteristics and root exudate content (pH, TOC, TN, soluble sugars, and organic acids), and increased the absorption and accumulation of Cd by *S. alfredii*. Elevated CO_2_ levels were observed to promote plant growth by improving the photosynthetic carbon absorption rate and photosynthetic light energy utilization efficiency, and the total Cd absorption in the stems of *S. alfredii* increased by 44.1% to 48.5% [84].

In addition to risks to human health, there is potential for soil contamination when nanoparticles are applied to plants or soil. Agricultural soil can be the most prominent sink for nanoparticles, as portions of nanoparticles are likely to be retained in the soil, which may influence soil enzymatic activity and soil microbiota. Nanoparticles’ accumulation in soil may harm the soil ecosystem. Also, some types of nanoparticles are highly mobile in soil and can easily leach into groundwater or surface water systems [85,86]. This must be carefully managed, and the long-term effectiveness and environmental safety, as well as the risk of nanoparticle application in various soil ecosystems and plant species, should be evaluated. We could mitigate these risks and harness the benefits of these innovative technologies while protecting the environment and human health by establishing robust regulatory frameworks. While the potential benefits of CO_2_ enhancement in agriculture are promising, several practical considerations must be thoroughly evaluated before widespread implementation. One of the primary concerns regarding CO_2_ enhancement is the economic cost associated with its application, which requires significant infrastructure investment and ongoing operating expenses. The balance between input costs and output benefits needs to be considered. The economic feasibility of CO_2_ enhancement varies depending on the scale of operation, regional market conditions, and government subsidies or incentives. Additionally, the integration of CO_2_ enhancement strategies into existing agricultural practices necessitates careful consideration to ensure compatibility and minimize disruptions. Traditional farming systems, particularly in developing countries, often rely on low-tech, labor-intensive methods. Introducing complicated CO_2_ delivery systems could be logistically challenging and may require significant adaptations in farming workflows. Therefore, the implementation process needs to pay attention to cost control and coordination with other agricultural practices, as well as the long-term impact on the environment and the economy.

### 3.5. Integrated Approach-Assisted Phytoextraction

In practical applications, a single measure may be not enough for hyperaccumulator plants to effectively extract Cd. It is necessary to combine multiple measures to create a synergistic effect and enhance Cd removal efficiency. For example, integrated strategies such as the screening of highly efficient Cd hyperaccumulators, the optimization of agronomic measures, the improvement of soil environments, and the adoption of combined plant–microbe remediation could significantly improve the effectiveness of phytoextraction for Cd-contaminated soils. For example, the application of electric fields and soil amendments (such as pig manure compost, humic acid, and EDTA) can significantly increase Cd content in *S. alfredii* (*p* < 0.05). The combined measure of pig manure compost and direct current electric field achieved the best results, enhancing the Cd accumulation in shoots by 1.92 times [87]. Chen et al. (2024) [7] found that the growth and Cd extraction efficiency of *S. alfredii* were enhanced in both acidic and alkaline soils through the use of a synergistic system of foliar application combined with plant growth regulator BRs and PGPB (*P. fluorescens*), with significant increases in Cd removal rates of 355.2% and 155.3%, respectively, compared to the control. The combined treatment led to reductions in soil pH and DTPA-Cd content and an increase in soil enzyme activity, which, in turn, improved the soil microecology and the diversity of rhizosphere microbial community structures. This treatment also facilitated the absorption and translocation of Cd in the aboveground tissues of *S. alfredii*. The study concluded that the synergistic application of BR and *P. fluorescens* could be a practical approach to enhance the phytoextraction effect of *S. alfredii* in various soil types. The combined application of EDTA and plant growth-promoting rhizobacterium Burkholderia sp. significantly increased Cd concentrations in *S. alfredii* plants and enhanced the levels of cysteine and phytochelatins [88]. When treated together with 50 μmol L^−1^ indole-3-acetic acid (IAA) and 2.5 mmol kg^−1^ oxalic acid, *S. alfredii* exhibited its highest biomass, and the synergistic effect led to an 82.4% increase in Cd phytoextraction. This effectively improved the remediation of Cd-contaminated soil [80]. Additionally, the combined approach of intercropping and inoculation presents a viable strategy to enhance Cd phytoextraction efficiency for plants, providing a comprehensive technical model for the simultaneous production and remediation of lightly to moderately Cd-contaminated soils. Cao et al. (2024) [44] demonstrated that intercropping *Brassica napus* and *S. alfredii*, along with the inoculation of PGPB, effectively enhanced plant growth and Cd accumulation across three different agricultural sites with Gleysol soil, stagnic anthrosols, and inceptisol in a subtropical monsoon climate, while the soil Cd concentration ranged from 0.28 to 0.85 mg kg^−1^. This synergistic treatment showed better capabilities in increasing biomass and Cd accumulation of both plants. When implementing such combined measures, it is crucial to consider the interactions and influences among the different treatments to avoid any negative effects, and a deeper exploration of the synergistic effects observed in integrated approaches would be valuable for Cd remediation. In summary, enhancing Cd phytoextraction through the use of hyperaccumulator plants using combined measures is a complex process that needs to consider multiple factors and appropriate strategies. By continuously optimizing these measures, the efficiency and effectiveness of phytoextraction for Cd-contaminated soils can be further improved.

However, as there may be potential conflicts between different strategies, it is important to consider their individual strengths, weaknesses, and potential interactions. For example, chemical amendments may alter the soil characteristics and affect the availability of essential nutrients [89], making them less favorable for certain microorganisms, thereby inhibiting microbial activity. Some chemical amendments may be toxic to microorganisms, either directly or indirectly through the formation of byproducts. Conversely, the metabolites produced by microorganisms during the biodegradation process may interact with chemicals in unpredictable ways, potentially leading to the formation of harmful compounds. It is possible to achieve effective and sustainable remediation outcomes by understanding these conflicts and optimizing the combination of strategies through the control of the timing of application, careful planning, monitoring, and adjustment.

## 4. The Problem, Potential Solutions, and the Selection of the Best Assisted Phytoextraction Method

Despite the immense potential demonstrated by Cd hyperaccumulators and their enhancement strategies in removing Cd contamination from soil, the environmental trade-offs and socio-economic impacts that may arise from the large-scale application of these technologies cannot be overlooked. These include disturbances to soil ecosystems, long-term contamination of soil and groundwater, and the bioaccumulation of nanoparticles in the food chain, all of which could threaten human health. Furthermore, the implementation of the mentioned enhancement strategies often requires substantial initial investments, and their public acceptance may be influenced by various factors, such as low or even non-existent economic returns, public distrust of new technologies, and the lack of relevant regulations and standards. These factors could hinder the promotion and application of these technologies.

Moreover, post-harvest treatments and utilization of Cd-phytoextracted plant residues need to be investigated safely and economically, as the amount of straw derived from Cd hyperaccumulators is expanding. Multiple approaches have been reported, such as incineration, pyrolysis, and composting, alongside newer methods like phytomining and hydrothermal upgrading [90,91]. Among these, thermal treatments including incineration, gasification, and pyrolysis stand out due to their cost-effectiveness [92]. Cui et al. (2018) [93] suggested that gasification might be a promising method for the disposal of *S. alfredii* and the simultaneous production of multifunctional materials. Future research should concentrate on Cd-accumulated straw derived from phytoextraction under large field scales.

The successful promotion and large-scale application of assisted phytoextraction measures for Cd pollution may require policy support and market incentives from the government. For instance, providing financial subsidies, tax reliefs, or preferential loans could alleviate the economic pressure on enterprises and farmers in adopting new technologies. Additionally, the government could establish special funds to support research, development, and promotion with respect to soil remediation technologies, encouraging more enterprises and research institutions to engage in technological innovation. Secondly, stakeholder involvement is crucial for ensuring the successful promotion and implementation of these technologies. Participants need to jointly formulate plans and schemes for technology application. The public’s awareness and trust in new technologies can be strengthened by organizing expert lectures, on-site demonstrations, technical training, etc. At the same time, farmers and enterprises should be encouraged to actively participate in soil remediation efforts. Lastly, raising public awareness and attention with respect to soil pollution issues is an important way of promoting these strategies. The government can increase awareness of the hazards of soil pollution and the importance of remediation technologies through media campaigns, public benefit activities, and similar initiatives. Furthermore, a public participation mechanism can be established to encourage the public to participate in the supervision and evaluation of soil remediation projects [94]. Therefore, all parties, including the government, research institutions, enterprises, and farmers, should strengthen cooperation and communication to explore and implement more efficient, environmentally friendly, and economically feasible methods for soil remediation.

The same enhancement measures for a particular plant cannot be universally applied worldwide. There are many factors that influence the efficiency of assisted phytoremediation strategies, including climate type, soil type, and plant species. Thus, it is necessary to establish enhanced remediation models tailored to local conditions. The currently available assisted phytoextraction technologies discussed above for Cd-contaminated soil exhibit their own advantages and limitations, as summarized in Table 3. The applicability of these technologies in specific soil remediation projects mainly depends on factors such as the geographical location of the contaminated site, pollution characteristics, remediation goals, financial budget, degree of implementation readiness, cost-effectiveness, time requirements, and public acceptance. All these factors must be considered and comprehensively assessed to select the most suitable technology for a specific soil. In most regions, governments primarily rely on expert decision making, combined with the wishes of farmers, to develop several targeted plans and detailed remediation schemes after achieving a comprehensive and in-depth understanding of the soil structure, soil type, and heavy metal pollution characteristics in the area. Then, the feasibility of pre-screened assisted phytoextraction strategies is assessed by treatability studies [95], which is beneficial in selecting the most feasible phytoextraction technology.

In short, the scalability and practical implementation of enhanced strategies for Cd remediation in diverse agricultural systems require a comprehensive approach that considers cost, resources, technical expertise, regulatory frameworks, and context-specific conditions. Successful implementation depends on collaboration between government agencies, research institutions, farmers, and agricultural extension workers. Through continuous monitoring and evaluation, the effectiveness of these strategies can be assessed and adapted to changing conditions, ensuring the sustainability and safety of agricultural production in Cd-contaminated areas.

## 5. Conclusions

As a cost-effective and efficient technique, phytoextraction has gained significant attention for the management of Cd contamination in arable soil. While the theoretical framework for the use of Cd hyperaccumulator plants to address Cd contamination problems prevailing in agricultural soils is relatively well-established, there are still several challenges in the practical application of this technology in the field. To improve the effectiveness and efficiency of phytoextraction, it is essential to enhance the environmental adaptability of Cd hyperaccumulator plants. This includes expanding their suitable growth areas and developing a diversity of high-biomass species with a strong capacity for Cd accumulation. In addition to strictly adhering to threshold standards, it is essential to consider other hyperaccumulation characteristics and the complexity of soil pollution to screen for hyperaccumulator resources that are tolerant to multiple heavy metals and have strong adsorption capabilities. To promote the widespread adoption and application of phytoextraction using Cd hyperaccumulators, it is essential to enhance the study of rhizosphere microecological changes during phytoextraction. Additionally, there should be increased research and application of combined remediation technologies, with a focus on understanding how their synergistic effects enhance effectiveness. Furthermore, the full utilization of agricultural scientific management methods is needed to improve phytoextraction capabilities. Exploring plant-based remediation technologies that align with sustainable development principles has become a top priority in current phytoextraction research. The goals are to reduce the secondary environmental burden associated with combined remediation strategies and to minimize the impact of each soil remediation method on soil structure and quality. Furthermore, there is a pressing need to enhance research and development efforts focused on safe disposal and resource reuse technologies for Cd hyperaccumulators. Given the unique social and economic background, we must develop efficient, low-energy-consuming, non-secondary-polluting, and resource-renewable plant-based remediation methods.

In summary, the applicability of assisted phytoextraction technologies is influenced by numerous factors, including site and contamination characteristics, remediation goals, remediation efficiency, cost-effectiveness, time, and public acceptability. Due to the diversity of soil types and contamination sources, as well as the heterogeneity of spatial variation in Cd-polluted soils, it is challenging to generalize a universally applicable and cost-effective method. Therefore, it is necessary to develop locally suitable measures to make management more precise and effective. Treatability studies should also be conducted before full-scale remediation implementation to help select the most feasible strategy. In the future, more long-term field trials are needed to make these technologies commercially viable and acceptable. In-depth research into the mechanisms of assisted phytoextraction technologies will lay the foundation for theoretical work in phytoextraction. Moreover, the appropriate combination of existing remediation methods to achieve optimal heavy metal removal with minimal side effects is a key scientific research area for future soil remediation.

## Figures and Tables

**Table 2:** Comparison of effectiveness between Cd hyperaccumulators and crops under various planting patterns with different soil types and climatic conditions.

Planting Pattern	Plants	Soil Type	Climatic Conditions	Increase Rate %	References
Intercropping	*Sedum plumbizincicola*–corn	Typic agri-udic ferrosol	Moist monsoon climate	35.4	[64]
Intercropping	*Sedum alredii*–favabean	Gleysol soil	Subtropical monsoon climate	268	[55]
Relay cropping	*Solanum nigrum* L.–oilseed rape	Paddy soil	North subtropical monsoon climate	35.1	[56]
Relay cropping	*Sedum alredii*–soybean	Paddy soil	South subtropical climate	60.7	[65]
Rotation	*Sedum plumbizincicola*–rice	Anthropogenic hydric soil	Central subtropical monsoon humid climate	48.5	[62]

Note: The increase rate represents the percentage increase in plant Cd phytoextraction absorbed by hyperaccumulators under intercropping, relay cropping, or crop rotation compared with monoculture.

**Table 3:** Advantages, limitations, and applicability of the available enhanced remediation techniques for Cd-contaminated soils.

Enhancement Strategy	Advantages	Limitations	Applicability
Microbially assisted phytoextraction	Low cost and simple to implement	Lack of high efficiency and specificity for combined contaminants, and effectiveness depends on the type of soil, plant, and metals	In situ at contamination sites with low to moderate pollution and suitable soil environments
Agronomic measure-assisted phytoextraction	Easy to implement, low cost, environmentally friendly, and minimal soil disturbance	Time-consuming and low efficiency	In situ with low to moderate contamination; appropriate agronomic measures can be developed based on soil and plant characteristics
Chelate-assisted phytoextraction	High efficiency and fast effects	High cost, secondary pollution, and potentially toxic to the environment	Suitable for sites with heavy pollution requiring quick extraction
Nanotechnology-assisted phytoextraction	Precise extraction and recycling of heavy metals	High cost, safety and environmental risks, and potential leaching into water systems	Suitable for sites that require high extraction efficiency and accuracy
CO_2_-assisted phytoextraction	Beneficial to plants and minimal soil disturbance	High cost, limited to small areas, and not compatible with existing agricultural practices	Suitable for phytoextraction under greenhouse or controlled environmental conditions
Integrated approach-assisted phytoextraction	High efficiency and reduced time of remediation	Implementation may involve multiple steps and techniques, and the operation is relatively complex	Sites with serious pollution requiring efficient phytoextraction
